# Mental Health Gender Gap of Gay and Lesbian Aging Adults: The Role of Age Discrimination and Internalized Homophobia

**DOI:** 10.1093/geroni/igaf122.2404

**Published:** 2025-12-31

**Authors:** Ella Cohn-Schwartz, Sigal Gooldin, Yaacov G Bachner

**Affiliations:** Ben Gurion University of the Negev, Be’er Sheva, HaDarom, Israel; The Israeli Institute for Gender and LGBT Research, Tel Aviv, Tel Aviv, Israel; Ben Gurion University of the Negev, Be’er Sheva, HaDarom, Israel

## Abstract

Gender disparities in mental health among older adults from sexual minorities remain underexplored. This study investigated how stigma related to age and sexual orientation - both enacted by others (discrimination) and internalized – is associated with mental health outcomes among lesbian and gay adults aged 50+. The study examined 411 adults aged 50 + (range: 50-85) from Israel who self-identify as lesbian women or gay men, via online questionnaires. Participants answered questions about experiencing discrimination based on their age and sexual orientation, about their internalized ageism and internalized homophobia, and about their mental health (depressive symptoms, anxiety, loneliness, and life satisfaction). Mediation analyses examined the role of stigma based on age and on sexual orientation in the association between gender and mental health. The results showed that lesbian women reported better mental health compared to gay men. The gender difference in mental health was partially explained by gendered experiences of age-based discrimination and internalized homophobia: Women experienced less age-based discrimination and reported lower internalized homophobia, and these were related to better mental health in terms of lower depressive symptoms, lower anxiety, less loneliness, and higher life satisfaction. Thus, the greater mental health challenges faced by gay men in later life may stem, in part, from higher exposure to age-based discrimination and greater internalization of negative societal attitudes toward their sexual orientation—potentially reflecting the cumulative impact of lifelong stigma. These findings underscore the need for better interventions aimed at distinct needs and experiences of aging men and women from sexual minorities.

